# Editorial

**Published:** 1902-03-15

**Authors:** 


### EDITORIAL.

A RECENT number of the *British Journal of Dental Science* publishes in full the circular letter sent out by St. Luke's Hospital, of Niles, Michigan, offering for a small fee certain professional emoluments. By a curious oversight—or was it purposely done?—the name of the place "*Niles*," was left out from both places where it was printed in the original letter, and a dash rule takes its place. Just what the purpose of the editor may have been in making this omission is not made known by any editorial comment. Unfortunately it is capable of such construction, by those acquainted with the attitude of certain English dentists toward American educational institutions, as does not tend to increase our respect for the personal and professional integrity of those who seem to be continually seeking some cause for discrediting professional education in America. This incident seems like an unworthy, if not a malicious attempt to discredit the reputation of an educational institution, which has an honored and respected reputation for maintaining the highest educational standards known either in Europe or America. The inference to be drawn, is, that because an infamous law-evading corporation, organized and conducted for the sole purpose of enriching its owners, by appealing to the vanity and cupidity of the ignorant and mercenary, happens to have a temporary home in the same State, the University of Michigan, although more than a hundred miles distant, is implicated. The transactions of St. Luke's Hospital, even by insinuation, should not be credited to this or any other professional school in the State of Michigan. It is surprising that any one should be so ignorant of conditions in this country as to make so gross a blunder. If however it was done advisedly, it is, to say the least, a serious breach of professional courtesy, and such a proceeding as would not be countenanced by the "*yellowest*" of secular journals in this country. It seems to us that it calls for a suitable explanation.

THE latest addition to dental periodical literature is the *Dental News*, published in New York City, by the Stowe and Eddy Co. and edited by N. H. Stowe. The first number though small contains many short and interesting paragraphs and is very well worth reading. We wish it success.

